# Molecular Insights into the Interaction of Cathepsin D and Iron in Chronic Wound Healing: Exploring Therapeutic Potential and Mechanisms

**DOI:** 10.3390/biomedicines13030544

**Published:** 2025-02-21

**Authors:** María Rodríguez-Moreno, Isabel Legaz

**Affiliations:** Department of Legal and Forensic Medicine, Biomedical Research Institute of Murcia (IMIB), Regional Campus of International Excellence “Campus Mare Nostrum”, Faculty of Medicine, University of Murcia (UMU), 30100 Murcia, Spain; m.rodriguezmoreno@um.es

**Keywords:** cathepsin D, chronic wounds, extracellular matrix remodeling, iron dynamics, wound healing

## Abstract

**Background:** Chronic wounds, such as diabetic ulcers, often fail to progress through healing due to persistent inflammation, infections, and extracellular matrix (ECM) imbalances. Cathepsin D, an aspartate protease active in acidic environments, plays a pivotal role in wound healing by mediating inflammatory responses, ECM remodeling, and macrophage phenotype transitions. Its dysregulation, however, can impair healing, highlighting the need for targeted modulation of its activity. The aim of this study was to investigate the molecular interaction between Fe^2+^ and cathepsin D’s catalytic core and ionic zipper under physiological and acidic conditions to identify strategies to enhance tissue repair and accelerate the healing of chronic wounds. **Methods:** The molecular structure of active cathepsin D was obtained from the Protein Data Bank (PDB) and analyzed using UCSF Chimera. Molecular interactions between cathepsin D and ferrous ions (Fe^2+^) were studied, focusing on key residues (D33 and D231) and ionic zipper residues (E5, E180, and D187). **Results:** Our results showed that the active form of cathepsin D, a 96 kDa dimer, consisted of heterodimers with distinct amino acid chains, where residues D33 and D231 formed the active site, and E5, E180, and D187 constituted the ionic zipper. A functional pocket containing the conserved residues D33 and D231, essential for proteolytic activity, was identified. At physiological pH (~7.5), D33 exhibited the most potent interactions with Fe^2+^, with interaction energies of −7 × 10^17^ J at oxygen atoms of the carboxylate group (OD1) and α-carbon (CA) atoms, whereas D231 showed slightly lower energies of −6 × 10^17^ J at γ-carbon atom (CG) and CA atoms. At acidic pH (~4), E5 was the primary interacting residue, with the shortest distance to Fe^2+^ (2.69 Å), and showed stable interactions across several atoms, emphasizing its role in metal binding. **Conclusions:** pH conditions strongly influence the interaction of cathepsin D with Fe^2^. At physiological pH, residues D33 and D231 demonstrate robust and energetically efficient binding with Fe^2+^. At the same time, under acidic conditions, E5 emerges as the primary residue involved, potentially affecting the ionic zipper of cathepsin D. These insights provide a molecular foundation for targeting specific residues to modulate cathepsin D activity, presenting promising opportunities for therapeutic strategies aimed at improving chronic wound healing.

## 1. Introduction

Wound healing during therapeutic interventions faces considerable challenges that hinder the recovery and repair of damaged tissues [1,2,3]. Chronic wounds, such as diabetic ulcers and pressure sores, are particularly problematic, often remaining trapped in the inflammatory phase without progressing to the proliferation and remodeling stages [4]. This stagnation significantly delays healing and is further exacerbated by persistent infections, unresolved inflammation, insufficient angiogenesis, and imbalances in the extracellular matrix (ECM) [5]. Cathepsins (Cts) have been identified as lysosomal protease enzymes that cleave peptide bonds [6,7]. Cathepsin D degrades damaged or senescent proteins within lysosomes, contributing to cellular clearance and tissue health [8]. These enzymes are now recognized for functioning beyond the lysosome, exhibiting activity in various cellular compartments and even under non-acidic conditions [6,7]. Based on their structure and catalytic mechanisms, cathepsins are categorized into three families: serine (e.g., cathepsins A and G), aspartate (e.g., cathepsins D and E), and cysteine (e.g., cathepsins B, L, and K) [9]. Through precise and selective proteolysis, these proteases play critical roles in regulating biological processes by ensuring controlled degradation of specific substrates [10]. Among these enzymes, cathepsin D stands out for its pivotal role in wound healing [8]. As an aspartate protease, it relies on two aspartic acid residues in its active site to hydrolyze peptide bonds, performing optimally in the acidic environment of lysosomes [11]. However, cathepsin D operates outside lysosomes during wound healing [12]. At sites of inflammation or injury, the extracellular microenvironment often becomes acidic due to the accumulation of lactate and reactive oxygen species (ROS), conditions that support its activity beyond its natural compartment [9]. Furthermore, interactions with stabilizing molecules, such as heat shock proteins (HSPs) and cytokines, enable cathepsin D to remain functional, even under suboptimal pH conditions [13].

Cathepsin D plays a dual role in wound healing, participating in the inflammatory and resolution phases [9,14,15]. During the acute inflammatory phase, it facilitates activating and releasing proinflammatory cytokines like IL-1β and TNF-α, amplifying the immune response to injury [16,17]. Simultaneously, it aids in the apoptosis of damaged or infected cells, a vital mechanism for limiting inflammation and clearing necrotic tissue from the wound site [18]. In the resolution phase, cathepsin D’s activity shifts to ECM degradation, promoting tissue remodeling and repair [15,19]. It regulates macrophage function by inducing their transition to an anti-inflammatory phenotype, crucial for resolving inflammation and restoring tissue homeostasis [20,21]. Throughout wound healing, immune cells like macrophages release cathepsin D to break down damaged ECM components and remove necrotic cells [22]. This proteolytic activity is indispensable for tissue remodeling in later healing stages, ensuring the formation of functional scar tissue through controlled degradation of collagen and other ECM proteins [15,23]. However, the dysregulation of cathepsin D’s activity can disrupt the balance of protein degradation, impairing the quality and pace of the healing process [24,25]. Metal ions, particularly iron (Fe), play a complementary role in wound healing by transitioning between reduced (Fe^2+^) and oxidized (Fe^3+^) states as the environment evolves from acidic and hypoxic in the early stages to more oxygenated and neutral during tissue repair [26,27,28]. Iron concentrations increase proportionally with the severity of vital injuries, emphasizing its critical role in cellular mechanisms mediating the response to tissue damage. Similarly, cathepsin D exhibited markedly elevated expression in vital wounds, which sets it apart from the patterns observed in undamaged tissues [29,30,31].

The aim of this study was to investigate the molecular interaction between Fe^2+^ and the catalytic core and ionic zipper of cathepsin D under physiological and acidic pH conditions. We examined how this relationship influenced the enzyme’s proteolytic activity to identify possible strategies to modulate its activity, improve tissue repair, and accelerate the healing of chronic wounds.

## 2. Materials and Methods

### 2.1. Molecular Structure of Cathepsin D

The Protein Data Bank (PDB ID: 1LYB) provided the tertiary structure of cathepsin D. The three-dimensional structure was determined through X-ray crystallography, with resolutions of 1.80 Å for the active form. It was analyzed and visualized using UCSF Chimera software (version 1.17.3), which enabled detailed comparisons to identify key differences in their three-dimensional configurations, particularly in the catalytic regions, and the ionic zipper [10] was used for three-dimensional visualization of molecular structures. Chimera software was used to analyze the docking results and to prepare the figures for the docked conformations. Before analysis, all non-protein atoms were removed from the PDB file. The protein structure was used for docking and prepared by incorporating all hydrogen atoms and eliminating water molecules.

### 2.2. Active Site Prediction, Functional Pockets, and Ionic Zipper in Cathepsin D

To predict and visualize the functional sites in active cathepsin D, the PrankWeb tool [32] was utilized. The server prioritizes pockets according to combined parameters, such as conservation, amino acid residues in every pocket, and probability score, then ranks the pockets accordingly.

### 2.3. Molecular Distances and Interaction Energies

The residues and atoms involved in the residues D33 and D231 were previously identified based on the structure obtained from the PDB. UCSF Chimera software (version 1.17.3) measured the distances between atoms and interactions, including only interactions <5 Å, classified as weak interactions (>4.0 Å), hydrogen/polar bonds (2.5–3.5 Å), and metal coordination (2.0–2.5 Å). The interactions identified included the name of the residue, the chain to which it belongs, the residue identifier, the specific atom involved, and the distance in angstroms (Å).

Interactions between active cathepsin D residues and ferrous (Fe^2+^) ions were analyzed. Interaction energies were calculated using AutoDock4.2.6 software and its complementary tool, AutoDockTools, and expressed in Joules (J) [33]. The scoring function in AutoDock is based on the United Atom version of the AMBER force field. In all cases analyzed, the dielectric constant of the environment was 80 for the aqueous medium.

## 3. Results

### 3.1. Molecular Characterization of Cathepsin D

**Figure 1:**
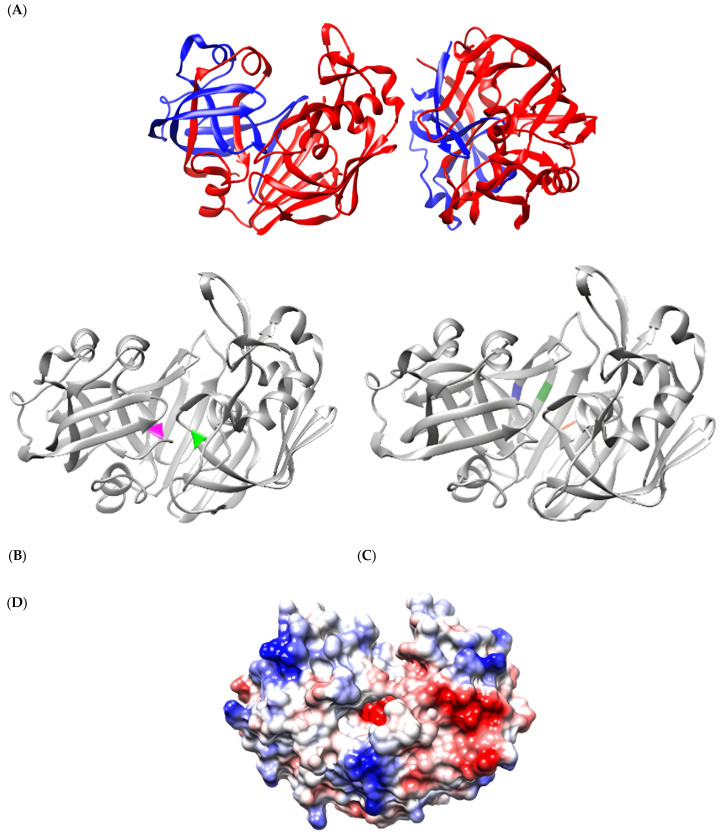
Molecular structure of active cathepsin D. (**A**) The dimeric molecular structure of active cathepsin D. The short chains (**A**,**C**), each composed of 97 amino acids, are shown in blue, while the long chains (**B**,**D**), each containing 241 amino acids, are represented in red. (**B**) The monomeric molecular structure of active cathepsin D highlights the active site of aspartic protease, with residues D33 and D231 marked in magenta and green, respectively. (**C**) The location of the pH-dependent regulatory ionic zipper, composed of residues E5 (blue), E180 (forest green), and D187 (orange). (**D**) Surface hydrophobicity analysis of active cathepsin D. The regions with strong negative charges (−10.0 kcal/mol e) are shown in red, neutral areas with no net charge (0.0 kcal/mol e) in white, and regions with strong positive charges (+10.0 kcal/mol e) in blue.

Active cathepsin D is a dimer of 96 kDa consisting of two identical heterodimers (Figure 1A). Each heterodimer comprises two distinct chains, chains A and B, which differ. In contrast, the second heterodimer comprises chains C and D. Tertiary structure analysis showed 27 β-sheets and seven α-helices. Chains A and C were identical (colored in blue), contained 97 amino acids, and had a molecular weight of 14 kDa. Similarly, chains B and D (colored in red) contained 241 amino acids, with a molecular weight of 34 kDa. The aspartate protease active site (residues D33 and D231) is in a short chain (blue) and a long chain (red), respectively (Figure 1B). The three amino acids (E5, E180, and D187) that constitute the ion zipper are in a short chain (E5) and long chain (E180 and D187) (Figure 1C).

Surface hydrophobicity analysis of cathepsin D showed a volume of 41.79 × 10^3^ Å^3^ and a surface area of 13.61 × 10^3^ Å^2^. The most hydrophobic regions were observed to be mainly distributed in areas close to the active site (D33 and D231) (Figure 1D).

**Figure 2:**
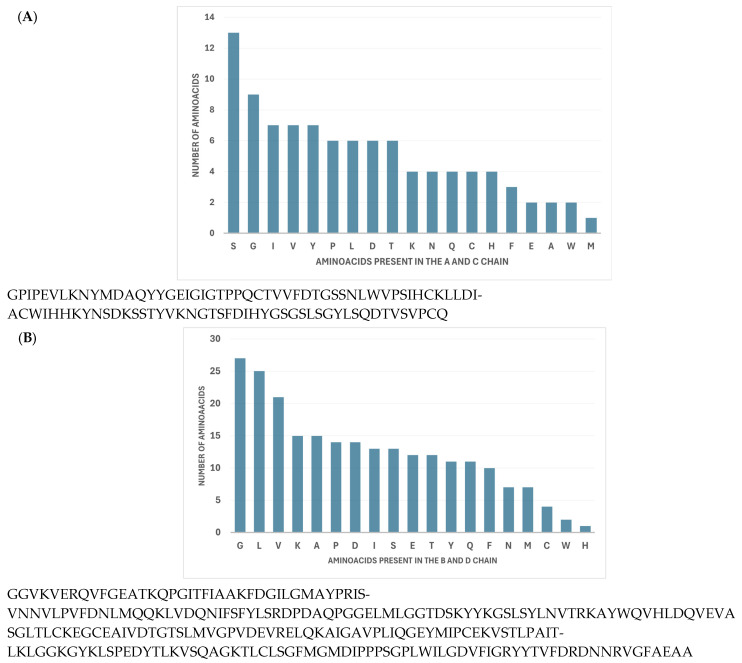
Analysis of the amino acid composition of cathepsin D. (**A**) The relationship and quantification of the amino acids present in the A and C chains of active cathepsin D. (**B**) Relationship and quantification of the amino acids present in the active form of the B and D chains in active cathepsin D.

As shown in Figure 2A, the most frequent amino acid in the A and C chains was serine (S, 13.4%), followed by glycine (G, 9.3%). The amino acids with less representation were glutamate (E, 2.1%), alanine (A, 2.1%), tryptophan (W, 2.1%), and methionine (M, 1.1%). Then, the most frequent amino acids in the B and D chains were glycine (G, 27.9%), leucine (L, 25.8%), and valine (V, 1.7%) (Figure 2B).

### 3.2. Analysis of Active Site Prediction, Functional Pockets

**Figure 3:**
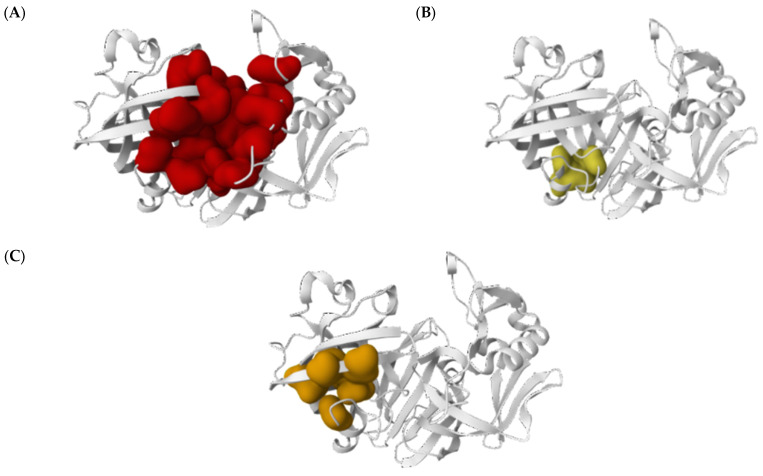
Molecular structure of active cathepsin D showing the presence of three functional pockets. (**A**) A structural representation of cathepsin D, focusing on chains C and D for molecular analysis. (**B**) Pocket 1, identified as the most probable functional site, is highlighted in red. This pocket includes residues D33 and D231, which are essential for the protease activity of cathepsin D. (**C**) Pocket 2 is shown in yellow, and Pocket 3, is marked in orange, representing the additional regions with potential functional relevance based on their molecular interactions.

Functional pocket prediction analysis of cathepsin D showed the presence of three functional pockets (Figure 3).

The results showed that pocket 1 obtained a score of 29.18 and a functional probability of 91.2%, indicating that it was the most probable active site of the three detected. This pocket comprises 33 residues, among which D33 and D231 are essential active site components. The average evolutionary conservation of this pocket is 1669, which supports its functional relevance (Table 1). On the other hand, Pockets 2 and 3 presented lower scores and lower functional probability (3.2% and 1%, respectively) but could be involved in specific molecular interactions. Pocket 2 includes five amino acids, and Pocket 3 contains seven amino acids.

The position of the amino acids in the protein was then analyzed for each pocket predicted (Table 2).

### 3.3. Analysis of Molecular Interactions Between Active Cathepsin D and Fe^2+^ Under Physiological pH Conditions (~7.5)

The analysis of molecular interactions between the Fe^2+^ and the amino acids in the active form of cathepsin D showed a total of 9 amino acids involved (F32, D33, T34, G35, D231, T232, G233, T234, and D323), of which D33 and D231 were involved in the proteolytic activity of cathepsin D (Table 3).

The analysis of molecular interactions between D33 and Fe^2+^ showed six interactions with distances between 3.29 and 4.29 Å. The closest interaction (3.29 Å) was established between the carboxylic group (OD1) and the metal ion, indicating a significant interaction, probably through hydrogen bonds. On the other hand, D231 showed five interactions with Fe^2+^ with distances between 3.37 and 4.69 Å, which positions it within the range of weak interactions. The closest interaction (3.37 Å) was the one established with the carboxylic group (OD1).

### 3.4. Analysis of the Interaction Energies of D33 and D231 with Fe^2+^ Under Physiological pH Conditions (~7.5)

**Figure 4:**
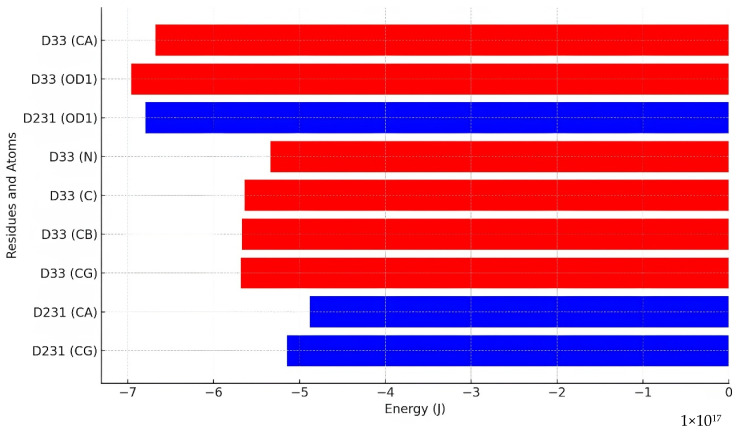
Comparison of the interaction energies between residues D33 and D231 of cathepsin D with Fe^2+^ under physiological pH conditions (~7.5). The red bars represent D33, while the blue bars correspond to D231, illustrating variations in interaction energies depending on specific atomic interactions. The lowest energy values are observed at the oxygen atoms of the carboxylate group (OD1) and the α-carbon (CA) in D33, reaching approximately −7 × 10^17^ J, indicating strong and stable interactions. In contrast, D231 exhibits slightly less intense interaction energies, with the γ-carbon (CG) and α-carbon (CA) showing values close to −6 × 10^17^ J, suggesting a lower but still significant affinity for Fe^2+^.

The interaction energies of D33 and D231 with the Fe^2+^ are shown in Figure 4. The lowest energies correspond to the interactions of D33, specifically at the oxygen atoms of the carboxylate group of the side chain (OD1) and α-carbon (CA) atoms, reaching values close to −7 × 10^17^ J. These negative energies suggest a strong and stable interaction at these points, which may be relevant for bond formation or electrostatic interactions. On the other hand, D231 shows significant negative energies but is slightly less intense than D33. In particular, the γ-carbon (CG) and CA atoms of D231 show values close to −6 × 10^17^ J, indicating that it also actively participates in the interactions, although with a lower magnitude of energy.

### 3.5. Analysis of D33, D231 E5, E180 and D187 Interaction Energies with Fe^2+^ Under Acidic pH Conditions (~4)

**Figure 5:**
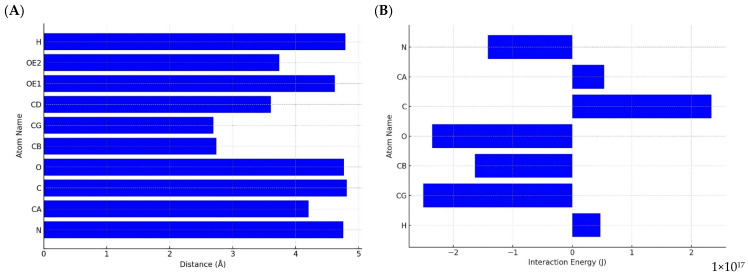
Analysis of the interaction between residue E5 (ionic zipper) of cathepsin D and Fe^2+^ under acidic pH conditions (~4). (**A**) Distance analysis of E5 interactions with Fe^2+^, showing that the shortest and strongest interaction occurs at the γ-carbon (CG) with 2.69 Å, followed by a secondary interaction at the β-carbon (CB), with 2.74 Å. These findings suggest a key role of E5 in metal affinity under acidic conditions. (**B**) A comparison of the interaction energies for E5 with Fe^2+^, highlighting variations in interaction strength depending on the specific atomic contacts.

The interactions between Fe^2+^ and protein residues focused on the catalytic core (D33, D231) and ionic zipper (E5, E180, and D187) were analyzed under acidic pH conditions (~4). The analysis identified the E5 residue as the only one that showed significant interactions with Fe^2+^. This residue established contact with the metal through several atoms, including amino group (N), α-carbon (CA), carboxyl group (C), oxygen atoms (O), β-carbon (CB), and, especially, γ-carbon (CG), which presented the shortest distance of 2.69 Å, indicating a stronger and more direct interaction. On the other hand, the CB, although close, showed a slightly larger distance of 2.74 Å, positioning itself as a secondary interaction point (Figure 5). These interactions highlight the importance of E5 in metal affinity, especially in an acidic environment where the protonation of carboxylate groups plays a key role in stabilizing these interactions. In contrast, the D (23, 33, and 187) and E180 residues did not show detectable interactions with Fe^2+^ within the range considered (≤5 Å).

## 4. Discussion

This study investigated the molecular interaction between Fe^2+^ and the catalytic core and ionic zipper of cathepsin D under physiological and acidic pH conditions. It examined how this relationship influences the enzyme’s proteolytic activity to identify possible strategies to modulate its activity, improve tissue repair, and accelerate the healing of chronic wounds.

Cathepsin D is essential for lysosome protein degradation and regulates apoptosis, extracellular matrix remodeling, and immune response [11]. Its expression and activity are finely regulated, and alterations in its levels may contribute to various diseases, including neurodegenerative disorders and cancer. Maintaining an adequate balance in cathepsin D activity is necessary to ensure cellular homeostasis and prevent pathologies associated with deregulation [8]. Further, cathepsin D is an aspartyl protease active in acidic environments and plays a crucial role in the different phases of wound healing, from initial inflammation to tissue remodeling [15,34]. Other studies [29,31,35] showed that cathepsin D expression was significantly increased in vital wounds, such as ligature marks in hanging deaths, compared to undamaged tissue [1,2,3]. This increase could be attributed to its central role in extracellular protein degradation and tissue remodeling during early inflammation. Its elevated activity under these conditions highlights its importance in the early stages of the tissue repair process. Iron concentration was observed to increase vital injuries, establishing an essential link between Fe^2+^ levels and cathepsin D activity [29]. The increase in Fe^2+^ in wounds is mainly due to hemorrhage; however, the inflammatory response plays a crucial role in iron mobilization [36]. During this phase, macrophages release iron stored in ferritin to enhance their antimicrobial capacity. This iron generates reactive oxygen species (ROS) essential to fight infections [37,38,39]. In addition, inflammation can activate metalloproteins that mobilize iron from intracellular stores, further increasing Fe^2+^ levels in the environment [40]. Hypoxia, a common feature in wounds, also contributes to the increase in Fe^2+^. Under low oxygen conditions, iron tends to be reduced from its ferric form (Fe^3+^) to its ferrous form (Fe^2+^) [41]. This process is favored by anaerobic metabolism that generates lactate, which acidifies the medium and improves the solubility of ferrous iron [42]. Our results show that at physiological pH, cathepsin D, in the context of the initial phase of a wound, could see its protease function compromised by the binding of Fe^2+^ to the two amino acids involved in its proteolytic activity when it is released from the interior of the lysosome into the extracellular medium. This interaction could affect its capacity to participate in the degradation of the extracellular matrix and the resolution of inflammation. Then, during the inflammatory phase of healing, cathepsin D facilitates the activation of proinflammatory cytokines, such as IL-1β and TNF-α, and promotes apoptosis of damaged or infected cells, allowing for the clearance of injured tissue [6]. This inflammatory environment is characterized by an acidic pH supporting optimal cathepsin D activity [11]. During this phase, iron accumulation, driven by hemoglobin release and macrophage activity, has the potential to influence the enzyme’s functionality by interacting with its catalytic residues (D33 and D231) and ionic zipper residues (E5, E180, and D187). However, our findings reveal that pH variation significantly alters these molecular interactions, with only E5 exhibiting binding to iron under acidic conditions, while D33 and D231 remain unaffected.

Our findings suggest that, under physiological conditions (~pH 7.5), Fe^2+^ inhibits cathepsin D by binding to the catalytic residues D33 and D231, interfering with its proteolytic activity. However, at acidic pH (~4), characteristic of inflammatory environments, this interaction disappears, and Fe^2+^ instead binds to E5, a key residue of the enzyme’s ionic zipper. This binding could induce conformational changes that alter its function differently from pepstatin A, which directly inhibits cathepsin D by binding specifically to D33 and D231 at any pH. While pepstatin A blocks enzymatic activity, directly decreasing its proteolytic capacity [43], Fe^2+^ at acidic pH may modify the enzyme’s structure and affect its function indirectly. It was observed that efficient inhibition of cathepsin D was achieved in biological experiments near neutral pH, with large molar excesses of pepstatin over cathepsin D [43]. Furthermore, Fe^2+^ binding to ionic zipper residues could destabilize the enzyme structure, affecting its role in extracellular matrix degradation and inflammation resolution. During the resolution phase, cathepsin D’s activity shifts toward tissue remodeling through the controlled degradation of extracellular matrix components. This process is essential for the formation of functional scar tissue. However, excess Fe^2+^ or pH imbalance could interfere with these functions, perpetuating inflammation or altering the quality of regenerated tissue. Chronic wound management includes advanced dressings, topical growth factor therapies, and nanotechnology-based technologies for controlled drug release. However, these approaches do not always address the specific molecular alterations that affect the biochemical environment of the wound, such as dysfunctions in cathepsin D activity.

The main limitation of our study was its reliance on molecular interactions through computational simulations, which may not fully reflect the complexities of the physiological environment. Future studies should address these limitations by confirming, in chronic wounds, the influence of modulating the ionic microenvironment using specific iron chelators to reduce its inhibitory impact on cathepsin D, as well as developing mimetic peptides capable of stabilizing the enzyme in the presence of iron.

## 5. Conclusions

In conclusion, our study revealed that the interaction between cathepsin D and ferrous ions (Fe^2+^) is highly influenced by pH conditions, with distinct residues playing pivotal roles under physiological and acidic environments. At physiological pH, D33 and D231 demonstrated a strong affinity for Fe^2+^, potentially inhibiting the proteolytic activity of cathepsin D and impairing its ability to degrade the extracellular matrix and resolve inflammation. Conversely, under acidic conditions typical of inflamed wounds, residue E5 emerged as the primary interaction site, suggesting a potential regulatory mechanism for the enzyme’s activity in such environments. These findings underscore the significance of the ionic microenvironment in modulating cathepsin D function and highlight opportunities for therapeutic interventions, including using iron chelators, mimetic peptides, and nanotechnological approaches targeting pH and iron states. Such strategies could enhance wound healing by optimizing the balance between inflammation and tissue remodeling phases.

## Figures and Tables

**Table 1:** Prediction and analysis of active pockets in cathepsin D: functional relevance and evolutionary conservation.

Rank	Score	Probability	# of Residues	Avg Conservation
1	29.18	0.912	33	1.669
2	1.79	0.032	5	2.013
3	0.56	0.001	7	1.339

Rank: the order of relevance of the predicted pockets. Score: the value that reflects the probability that a pocket is functional. Probability: the probability associated with the pocket. # of residues: the number of amino acids that make up the pocket. Avg conservation: the average degree of evolutionary conservation of the residues in each pocket.

**Table 2:** Amino acids are identified in the different pockets in active cathepsin D.

Pocket (Rank)	Identified Amino Acids
1 (Prob. 91.2%)	A13, Q14, V31, **D33**, G35, S36, S37, I76, H77, Y78, G79, S80, T125, F126, A129, F131, I134, I142, V144, Y205, I229, **D231**, G233, T234, S235, L236, Q260, V238, M307, M309, I311, S315, I320
2 (Prob. 3.2%)	M137, V150, F151, L154, L138
3 (Prob. 1%)	N38, L39, F74, I76, L87, G116, V147

Blue indicates that these amino acids belong to the short chain, and red indicates that they belong to the long chain. The amino acids in bold and underlined correspond to the protease activity of cathepsin D.

**Table 3:** Analysis of cathepsin D amino acids and Fe^2+^ interactions under physiological pH conditions (~7.5).

Residue	Chain	ID	Atom	Dist. (Å)	Interaction Type
F	C	32	C	4.34	Weak interactions
F	C	32	O	3.63	Other rank
D	C	33	N	4.29	Weak interactions
D	C	33	CA	3.43	Other rank
D	C	33	C	4.06	Weak interactions
D	C	33	CB	4.04	Weak interactions
D	C	33	CG	4.03	Weak interactions
D	C	33	OD1	3.29	Hydrogen/polar bonds
T	C	34	N	3.81	Other rank
T	C	34	CA	4.98	Weak interactions
T	C	34	OG1	4.21	Weak interactions
T	C	34	H	3.19	Hydrogen/polar bonds
T	C	34	HG1	3.77	Other rank
G	C	35	N	4.9	Weak interactions
G	C	35	H	4.12	Weak interactions
D	D	231	CA	4.69	Weak interactions
D	D	231	C	3.68	Other rank
D	D	231	O	3.97	Other rank
D	D	231	CG	4.45	Weak interactions
D	D	231	OD1	3.37	Hydrogen/polar bonds
T	D	232	N	2.9	Hydrogen/polar bonds
T	D	232	CA	2.45	Metal coordination
T	D	232	C	1.57	Other rank
T	D	232	O	2.37	Metal coordination
T	D	232	CB	2.81	Hydrogen/polar bonds
T	D	232	OG1	2.44	Metal coordination
T	D	232	CG2	4.28	Weak interactions
T	D	232	H	3.25	Hydrogen/polar bonds
T	D	232	HG1	2.27	Metal coordination
G	D	233	N	1.12	Other rank
G	D	233	CA	1.99	Other rank
G	D	233	C	3.35	Hydrogen/polar bonds
G	D	233	O	4.33	Weak interactions
G	D	233	H	1.5	Other rank
T	D	234	N	3.9	Other rank
T	D	234	H	3.61	Other rank
T	D	234	HG1	4.82	Weak interactions
D	D	323	OD1	4.95	Weak interactions

The blue color means part of the C chain. The red color means part of the D chain of cathepsin D. Å: angstrom. ID, position of the residue in the protein. Dist., distance. N, C, CB, CG, CA, and OD1 refer to specific atoms or groups in the amino acid structure. N, nitrogen atom of the amino group. C, the carbon atom of the carboxyl group. CB, β-carbon atom, which is attached to the α-carbon (CA) and is part of the side chain. CG, the γ-carbon atom, which is connected to the β-carbon and carries the functional groups. CA, α-carbon atom, to which the amino group (N), carboxyl group (C), hydrogen (H), and side chain (CB) are attached. OD1, oxygen atoms of the carboxylate group of the side chain.

## Data Availability

No new data were created or analyzed in this study.
